# Detailed Analysis of Diffuse Large B Cell Lymphoma Patients: A Single-Center, Retrospective Study

**DOI:** 10.1155/2013/908191

**Published:** 2013-07-30

**Authors:** Murat Ozbalak, M. Cem Ar, Nukhet Tuzuner, Ayse Salihoglu, A. Emre Eskazan, Seniz Ongoren Aydin, Zafer Baslar, Teoman Soysal, Yildiz Aydin, Anil Barak Dolgun, Onder Ergonul, Burhan Ferhanoglu

**Affiliations:** ^1^Division of Haematology, Department of Internal Medicine, Istanbul University, Cerrahpasa Medical Faculty, Cerrahpasa Cd., No. 181, Kocamustafapasa-Fatih, 34098 Istanbul, Turkey; ^2^Division of Haematopathology, Department of Pathology, Istanbul University, Cerrahpasa Medical Faculty, Istanbul, Turkey; ^3^Department of Biostatistics, Hacettepe University Medical Faculty, Ankara, Turkey; ^4^Department of Infectious Diseases, Koç University Medical School, Istanbul, Turkey

## Abstract

The aim of this single-center, retrospective study was to investigate the impact of rituximab, reconsider the validity of International Prognostic Index (IPI), and evaluate the prognostic role of the cell of origin (CoO) in a relatively young cohort. Three hundred twelve diffuse large B cell lymphoma patients (median age: 52) were included. Rituximab significantly improved the 3- and 5-year progression free survival (PFS) (70% versus 65% and 41% versus 36%, resp.; *P* < 0.001) but led only to a slight, insignificant increase in 3- and 5-year overall survival (OS) (71% versus 77.3% and %67 versus 74.5%, resp.; *P* = 0.264). In the young, low risk patient subgroup (aaIPI = 0&1; *n* = 129), rituximab improved 3- and 5-year PFS and OS rates (*P* < 0.001 and *P* = 0.048, resp.). The efficacy of rituximab in young high risk patients was comparable to the literature. CoO data were available in 190 patients. The OS at 3 years was 79% for GC and 64% for non-GC subgroups (*P* = 0.014). To the best of our knowledge, this is the first study which investigated the impact of R-CHOP in the context of CoO and IPI in a relatively young cohort. CoO was not an independent risk factor for prognosis in the multivariate analysis although patients with GC showed a significant survival advantage in the univariate analysis. CoO was also found to be a significant determinant of response in refractory/relapsed patients. Our results confirm the efficacy of rituximab in low and high risk, young patients outside of a randomized clinical trial setting.

## 1. Introduction

Diffuse large B cell lymphoma (DLBCL), being the most common morphological type, constitutes about 40% of newly diagnosed non-Hodgkin's lymphoma (NHL) cases. It is a heterogeneous disease with variable clinical course and prognostic outcome. Addition of the immunotherapeutic agent rituximab to chemotherapy improved the response rates in NHL [1]. However, despite major progress in the treatment, responses are not durable and the outcome is fatal in almost half of the patients with DLBCL. Therefore, great interest has been shown to develop prognostic scoring systems that would predict the outcome and identify patients with worst prognosis who would benefit from treatment strategies other than the standard regimens. 

Until recently, International Prognostic Index (IPI) [2] was almost the only widely used prognostic indicator in DLBCL. However, increasing evidence suggest that IPI fails to predict the prognosis in a considerable portion of patients with DLBCL. Consequently, there is a need for an improved and/or refined prognostic index which would predict the outcome more precisely. In search for such an index, various immunohistochemical markers have been investigated in DLBCL. Gene expression profiling studies identified three prognostically significant groups defined as “germinal center B cell (GCB),” “activated B cell,” and “primary mediastinal B cell” [3, 4]. High cost and technical requirements render gene expression studies impractical for routine use in most centers. To overcome this restriction Hans et al. proposed immunohistochemical methods which can differentiate GCB from nongerminal center B cell (NON-GC) DLBCL and efficiently replace gene expression profiling [5]. 

To detect the behaviour of the disease in young population, there are some clinical trials on the outcome of DLBCL in young cohorts [6]; however the data about real-life young patients are scarce. 

The aim of this single-center study was to investigate the impact of rituximab on the outcome of DLBCL in both low and high risk groups, to reconsider the validity of the International Prognostic Index (IPI), and to evaluate the prognostic role of the cell of origin (CoO) (“germinal center B cell-like (GC),” “activated B cell”) in a relatively young cohort of patient outside of a prospective clinical trial setting. 

## 2. Patients and Methods

### 2.1. Patients

All patients (*n* = 312) diagnosed with DLBCL according to the World Health Organization criteria [7] and followed at the Haematology Department of Cerrahpasa Medical Faculty, Istanbul University, from January 2000 to May 2011, were retrospectively included in this analysis. No preset selection criteria were defined for patient inclusion in the study except the pathologically confirmed diagnosis of DLBCL. The patient characteristics are given in Table 1. 

### 2.2. Ethics

The study was approved by the local ethics committee and conducted in accordance with the rules of Good Clinical Practice and Helsinki Declaration.

### 2.3. Definitions

Patients were staged according to Ann Arbor classification [8]. Complete response (CR), partial response (PR), progression, refractory disease, and relapse were defined according to ECOG criteria [9]. Any tumor mass measuring greater than 5 cm was accepted as “bulky disease” [10]*.*

### 2.4. Histopathological Analysis

Histopathological analysis of the lymph node materials was carried out at the Pathology Department of Cerrahpasa, Medical Faculty, by an expert hematopathologist. Identification of CoO as GC or non-GC was done according to the algorithm proposed by Hans et al. [5]. 

### 2.5. Statistical Methods

Statistical analyses were done with STATA/SE version 10.1 for Windows and R version 2.11.1. All patients, diagnosed with DLBCL before January 2004 (*n* = 54), were treated with CHOP (cyclophosphamide, doxorubicin, vincristine, and prednisolone) regimen. Following the approval of the reimbursement of rituximab in Turkey in December 2003, rituximab added CHOP or CHOP-like regimens became the standard first-line chemotherapy for DLBCL. The treatment cycles were applied every three weeks. In the study the patients were primarily assessed according to their response to the first-line treatment.

## 3. Results

A total of 312 DLBCL patients were retrospectively included. The cohort consisted of relatively young patients with a median age of 52 years. One-third of the patients had high risk characteristics (Table 1). Among the 190 patients analyzed for the CoO, 104 and 86 patients had GC and non-GC types, respectively. In the cohort, there were also 34 patients with T cell rich B cell lymphoma (TCRBCL) and 10 primary mediastinal large B-cell lymphomas (PMBCL). Twenty-two percent of patients had primary extranodal disease, most of which were primary gastrointestinal lymphomas (Table 2). High dose chemotherapy and autologous peripheral blood stem cell transplantation were performed in 25 patients at relapse (7 in GC, 6 in non-GC, and 12 in unspecified subgroup).

### 3.1. Survival Analyses

Survival data were updated as of June 2012. The Kaplan-Meier estimates of 3- and 5-year OS rates for the entire cohort were 76.2% (%95 CI: 0.71–0.81) and 73.1% (%95 CI: 0.67–0.78), respectively. Median follow-up period was 40 months (range, 1–142). PFS rates at 3 and 5 years were 64.5% (%95 CI 0.59–0.70) and 59.6% (%95 CI 0.53–0.65), respectively. Among patients who relapsed after having achieved CR with the first-line treatment, the median time to relapse (TTR) was 10 months (range, 1–53). Survival results according to the CoO are given in Table 3.

### 3.2. Comparison according to Treatment

First-line treatment resulted in CR in 248 patients (80%). PR was achieved in 10 patients (3%), whereas 1 patient had stable disease (SD) and 52 patients (17%) had progressive disease. Rituximab added to chemotherapy significantly improved PFS and ameliorated OS by 7%. The patient characteristics of the two treatment groups are detailed in Table 5 and the impact of rituximab on the survival rates is given in Table 3. 

### 3.3. Comparison according to CoO

The CoO was not an independent prognostic factor in our cohort of patients. The analysis of 190 patients with defined CoO resulted in similar patient characteristics apart from the LDH levels (Table 4).

**Figure 1:**
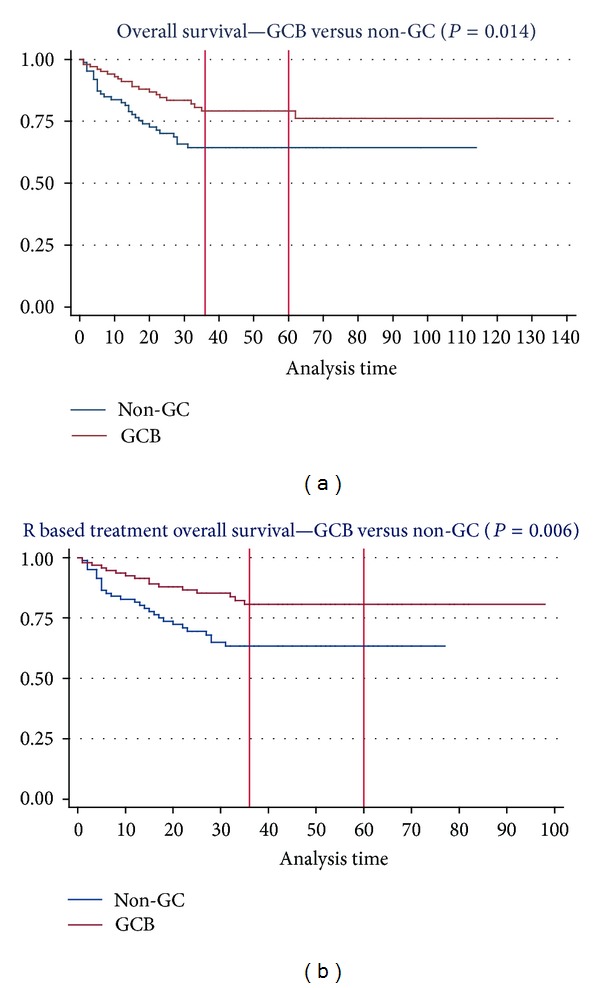
Kaplan Meier survival estimates—GC versus non-GC.

The OS rates at 3 and 5 years were both 79% (95% CI: 0.69–0.86) for GC and 64% (95% CI: 0.53–0.74) for non-GC subgroups (*P* = 0.014 median follow-up 38 versus 31 months, Table 3). We found lower mortality rates in the GC group (*P* = 0.023). GC type favoured a survival advantage and lower mortality rate even in patients treated with CHOP (*P* = 0.004). Three- and 5-year OS rates in patients who received R-CHOP were 81% (95% CI: 0.70–0.88) for GC and 63% (95% CI: 0.51–0.73) for non-GC group (median follow-up 38 versus 31 months, *P* = 0.006, Figure 1). On the other hand, the progression rates were similar in both groups (*P* = 0.181, Table 3).

Response to the first-line treatment in the GC group was significantly better than the non-GC group; the CR rates were 83% and 74%, respectively. Refractoriness to the first-line treatment was significantly higher in non-GC group (*P* = 0.012).

Among the 95 patients, who had primary refractory or relapse disease in our cohort, CoO results were only available for 67 patients. In this subgroup, CoO was the only significant determinant of progression (*P* = 0.003).

### 3.4. Parameters Affecting the Prognosis

**Figure 2:**
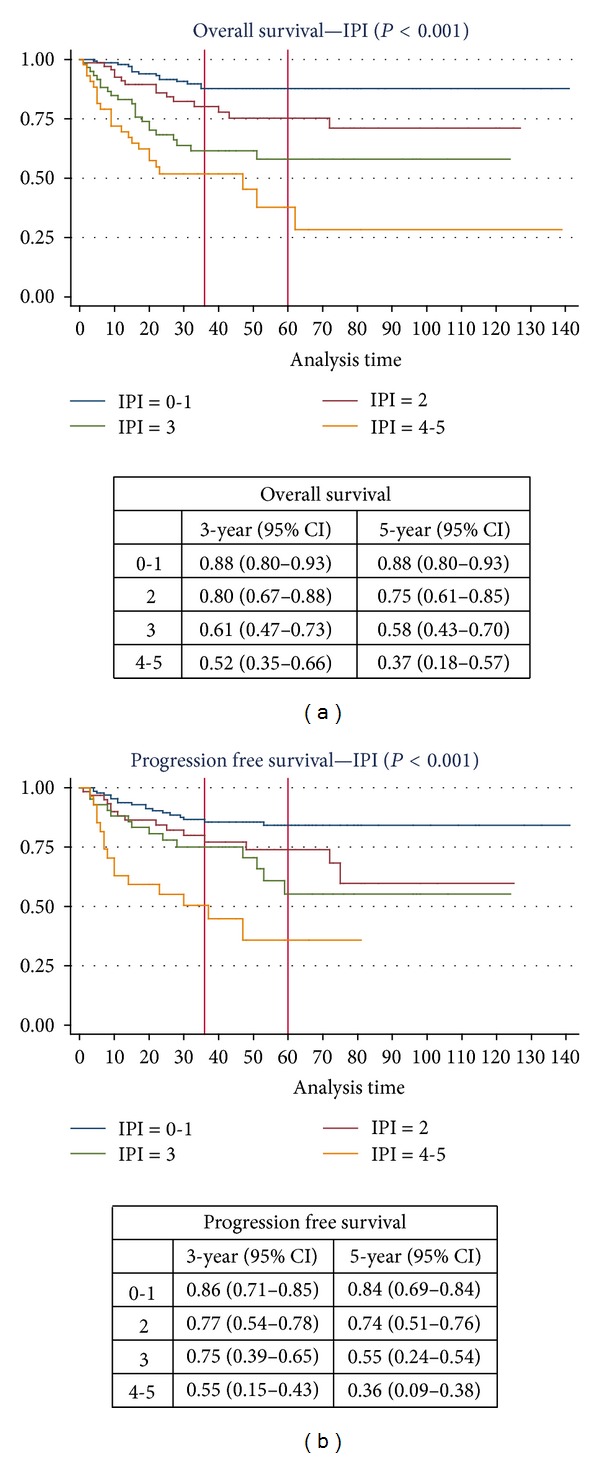
Kaplan Meier survival estimates according to the International Prognostic Index.

Univariate analysis highlighted advanced age, advanced stage of the disease, elevated lactate dehydrogenase (LDH) level, low performance status, and non-GC type as prognostic determinants (all *P* < 0.05). Extranodal involvement (>1 site), however, was not associated with clinical outcome (*P* = 0.276). This is in line with the original report [2] and can similarly be explained by the low median age (52 years) of our cohort. The OS and PFS rates according to IPI scores are shown in Figure 2. Among the 52 patients with primary refractory disease, all IPI parameters were found to be statistically insignificant (all *P* > 0.05) in the univariate analysis. 

We also did a subanalysis for prognostic outcome in the 190 patients with defined CoO. Advanced age, advanced stage, increased LDH levels, and low performance status significantly indicated a poor prognosis, whereas involvement of more than one extranodal site, diagnosis of primary extranodal lymphoma, and CoO were not associated with the prognosis (Table 6). Other parameters tested for prognostic outcome [11–16] (presence of B symptoms, liver involvement, spleen involvement, bone marrow involvement, bulky mass >5 cm, need for radiotherapy, extranodal involvement ≥3 sites, neutrophil/lymphocyte ratio >3.5, and absolute lymphocyte count <800/*μ*L) were insignificant. Initial thrombocyte level was available in 236 patients and a value less than 150 × 10^9^/L [17] was associated with increases in mortality (*P* = 0.013), bone marrow involvement (*P* = 0.009), splenic involvement (*P* = 0.016), and LDH level (*P* = 0.040) in univariate analysis, but it had no effect on prognosis in the multivariate analysis.

Of the 248 patients who initially achieved CR, 43 relapsed (median TTR: 10 months, range: 1–53). Involvement at more than one extranodal site (*P* = 0.001), advanced stage of the disease (*P* < 0.001), and elevated LDH level (*P* = 0.004) were associated with relapse in univariate analysis. In multivariate analysis, however, no risk factor was identified (all *P* > 0.05).

## 4. Discussion

Rituximab has been shown to be efficacious in the elderly as well as in the young low risk patients with DLBCL. Randomized studies on the effect of rituximab in newly diagnosed poor risk relatively young patients are scarce. These patients have also not been adequately represented in registry based studies. Consequently there is no consensus on how to treat this subgroup of patients. Young high risk patients have mainly been investigated in the setting of dose dense regimens and first-line high dose treatment with autologous stem cell support [18–20]. None of the studies showed a clear benefit in terms of overall survival. With the exception of MInT trial [6] there are no major rituximab studies on newly diagnosed young (<60 years) patients. Okamoto et al. newly published their retrospective data indicating improvement in the PFS of the patients older than 60 years in rituximab era [21]. Sehn et al. reported their results in rituximab era, comparing it with pre-rituximab era; however the median age of this population registry base study was 64 [22]. To the best of our knowledge, this study is one of the very few studies that evaluate the impact of rituximab on newly diagnosed DLBCL in a relatively young cohort outside of a clinical trial setting. 

Our cohort differed from the aforementioned rituximab studies in many aspects. First of all, it included a relatively young group of patients with a median age of 52 years. Furthermore, it included patients outside of a clinical trial protocol, that is, without any selection criteria. One-third of the patients had high risk disease according to IPI score.

Although we found similar response rates with R-CHOP and CHOP regimens, the 3- and 5- year PFS estimates in the R-CHOP and CHOP groups differed significantly. Our cohort was relatively small to predict overall survival difference. In our hands, rituximab significantly improved the PFS but the increase in OS was around 10%. 

Gene expression profiling has been reported to be an IPI independent prognostic marker [3]. Hans et al. [5] were the first to differentiate some surrogate groups by tissue IHC instead of gene expression profiling and reported a 5-year OS rate of 76% for GC and 34% for non-GC types. These results were confirmed by several other studies [23–26]. Thieblemont et al. demonstrated the influence of CoO in relapsed/refractory DLBCL patents [27]. Others, however, could not show the prognostic significance of CoO [28, 29]. 

In our single-centre retrospective analysis, CoO studies were done in 190 of the 312 patients (Table 4). LDH levels and mortality rates were significantly elevated in non-GC group, which might be explained by the aggressive behavior of this type (*P* = 0.023). The 5-year OS rates of the GC and non-GC groups were 76% and 64% (*P* = 0.014), respectively, which was in accordance with the current literature. The CR rates were 83% and 74% in the GC and the non-GC groups, respectively. Twelve percent of the patients in the GC and 24% in the non-GC group were refractory to the first-line treatment (*P* = 0.012). Moreover, the CoO was the only significant prognostic factor in the multivariate analysis of progressed patients (*P* = 0.003), with the non-GC being worse. 

Recently, several articles questioned the validity of IPI score in DLBCL in the context of rituximab [30]. However, we could demonstrate a clear stratification of OS and PFS rates in our cohort according to IPI score (Figure 2). Having extranodal involvement (>1 site) had no prognostic significance. Extranodal involvement (>1 site) was reported not to retain independent prognostic significance in patients younger than 60 years old in the original IPI study [2]. The lower median age (52 years old) in our cohort might explain this. IPI worked very well in our cohort of patients including those treated with rituximab. In opposition to the suggestion of Sehn et al. [31], we do not think that there is a need for the restratification of the IPI. But perhaps, the impact of the CoO on the IPI-predicted outcomes might be investigated in larger patient groups to see whether this helps to refine the definition of the patients with the worst prognosis.

The survival rates among the young, low risk patients (aaIPI scores 0 and 1) in our cohort were lower when compared to the MInT trial [6], but they differed significantly between treatment groups, being better in the R-CHOP group. Three- and 5-year OS rates were 90% for R-CHOP (*n* = 107) and 72% for CHOP (*n* = 22) groups (*P* = 0.048). Similarly, 3-year and 5-year PFS rates were 93% versus 53% and 91% versus 53% for R-CHOP and CHOP groups, respectively (*P* < 0.001).

To test the efficacy of rituximab in the young poor risk population we performed a subgroup analysis on the 71 young patients (age ≤ 60 years) with aaIPI scores of 2-3 in the cohort. R-CHOP versus CHOP comparison was not done since only 13 patients received CHOP based treatment. In the R-CHOP group, the 3- and 5-year OS were both 71%, and 3 and 5-year PFS rates were 75% and 64%. To compare our results with the literature, we analyzed the 2-year survival rates of R-CHOP group. Forty-five of the 71 young patients had an aaIPI score of 2. Their 2-year OS and PFS rates were 83% and 79%, respectively. Thirteen patients with an aaIPI score of 3 had 2-year OS and PFS rates of 59% and 75%, respectively. Vitolo et al. reported a 2-year PFS rate of %72 and an OS rate of 83% with high dose treatment in a similar population [32]. In another study, 2-year PFS rates in high-intermediate and high IPI groups were 66% and 75%, respectively, with corresponding OS rates of 70% and 82% [33].

Our single-center retrospective cohort study has some limitations and its results should therefore be cautiously interpreted. First of all, treatment groups were not stratified according to treatment dose intensity, Second, side effect analysis was not performed in the treatment groups. Third, the number of patients who received CHOP (*n* = 54) was small. This might be the underlying reason for the statistically insignificant improvement of OS. 

In conclusion, our results demonstrated that IPI still works and is valid in rituximab era outside of a clinical trial setting, in a cohort consisting of *relatively* young DLBCL patients. To our knowledge, this is the first study which investigated the impact of R-CHOP in the context of CoO and IPI in a *relatively* young cohort of patients. In this cohort, CoO was not an independent risk factor for prognosis in the multivariate analysis although patients with GC showed a significant survival advantage in the univariate analysis. CoO was an important and significant determinant of the response in refractory/relapsed patients. The survival rates among the young, low risk patients were lower when compared to the MInT trial, but they significantly differed between treatment groups, being better in the R-CHOP group. Rituximab was also effective in poor risk, young patients with intermediate/high risk factors.

## Figures and Tables

**Table 1:** Patient characteristics.

	All patients	Loss of the follow-up	Survivors	Dead	*P* value*
	*N* = 312	*N* = 30	*N* = 205	*N* = 77	
Female	143	17	96	30	0.123
Male	169	13	110	46	
Mean/median age (range)	51.3/52 (17–83)	54.9/55	48.3/47.5	58/60 (32–83)	0.104
Subgroup					
GCB	104	7	77	20	
Non-GC	86	7	49	30	**0.023**
Unknown	122	16	79	27	
Treatment regimen					
CHOP based	54	10	25	19	
R-CHOP based	258	20	180	58	**0.042**
Stage					
1	75	10	58	7	
2	66	4	50	12	
3	86	9	49	28	
4	85	7	48	30	**<0.001**
Age					
<60	208	19	151	38	
≥60	104	11	54	39	**<0.001**
LDH					
Normal	145	17	108	20	
High	162	13	93	56	**<0.001**
Extranodal					
<2	220	24	152	44	
≥2	92	6	53	33	**0.002**
Performance^*µ*^					
≥70	263	27	183	53	
<70	49	3	22	24	**<0.001**
BMI					
No	252	22	173	57	
Yes	57	6	32	19	0.090
Bulky mass (>5 cm)					
No	179	20	116	43	
Yes	129	9	87	33	0.754
IPI					
0-1	139	18	106	15	
2	68	5	48	15	
3	59	5	32	22	
4-5	43	2	18	23	**<0.001**
aaIPI					
0	67	7	56	4	
1	62	5	46	11	
2	53	4	37	12	
3	18	2	8	8	**0.002**
Primary extranodal					
No	243	22	153	68	
Yes	69	8	53	8	**0.005**

GC: germinal center B cell.

Non-GC: nongerminal center B Cell.

LDH: lactate dehydrogenase.

BMI: bone marrow involvement at initial diagnosis.

*Comparison between surviving and dead patients.

^*µ*^Karnofsky performance scale.

**Table 2:** Primary extranodal lymphomas.

Stomach	20
Ileum	4
Rectosigmoid junction	1
Tongue	1
Submandibular salivary gland	1
Liver	2
Spleen	1
Nasopharynx	2
Lung	2
Larynx	1
Tonsil	9
Thyroid	5
Musculoskeletal system	12
Leg	1
Ovary	2
Breast	1
Spinal cord	1
Paranasal sinus	2
Orbita	1

**Table 3:** OS and PFS rates in different subgroups according to CoO and treatment modalities.

	Median follow-up period	OS		PFS	
		3 years (95% CI)	5 years (95% CI)	3 years (95% CI)	5 years (95% CI)
GC Subgroup (*n* = 104)	38 months	0.79 (0.69–0.86)	0.79 (0.69–0.86)	0.70 (0.60–0.78)	0.61 (0.48–0.71)
Non-GC subgroup (*n* = 86)	31 months	0.64 (0.53–0.74)	0.64 (0.51–0.72)	0.61 (0.49–0.70)	0.61 (0.49–0.70)

Significance level		*P* = 0.014		*P* = 0.573	

R-CHOP Group (*n* = 258)	37 months	0.77 (0.71–0.82)	0.74 (0.68–0.80)	0.70 (0.64–0.75)	0.41 (0.27–0.54)
CHOP Group (*n* = 54)	60 months	0.71 (0.57–0.82)	0.67 (0.52–0.82)	0.65 (0.58–0.71)	0.36 (0.23–0.49)

Significance level		*P* = 0.264		*P* < 0.001	

**Table 4:** Comparison according to cell of origin.

	GC *N* = 104 (%)	Non-GC *N* = 86 (%)	*P* value
Female	42 (40)	43 (50)	
Male	62 (60)	43 (50)	0.330
Mean age	51	54.5	
Median age (range)	51 (20–81)	55 (26–80)	0.441
Treatment strategy			
CHOP	9 (9)	5 (6)	
R-CHOP	95 (91)	81 (94)	0.456
Radiotherapy			
Yes	25 (24)	14 (16)	
No	79 (76)	72 (84)	0.187
Stage			
1	28 (27)	20 (23)	
2	28 (27)	13 (15)	
3	27 (26)	31 (36)	
4	21 (20)	22 (26)	0.141
Age			
<60	69 (66)	52 (60)	
≥60	35 (34)	34 (40)	0.401
LDH			
Normal	60 (58)	37 (43)	
High	44 (42)	49 (57)	**0.044**
Extranodal			
<2	78 (75)	62 (72)	
≥2	26 (25)	24 (28)	0.651
Performance			
≥70	92 (88)	74 (86)	
<70	12 (12)	12 (14)	0.618
IPI			
0-1	55 (53)	34 (40)	
2	24 (23)	20 (23)	
3	14 (13)	16 (18.5)	
4-5	11 (11)	16 (18.5)	0.194

**Table 5:** Comparison according to the treatment.

	CHOP based *N* = 54 (%)	R-CHOP based *N* = 258 (%)	*P* value
Female	24 (44)	119 (46)	
Male	30 (56)	139 (54)	0.822
Mean age	47.8	52	
Median age (range)	46 (17–80)	53 (19–81)	0.183
Cell of origin			
GC	9 (36)	95 (54)	
Non-GC	5 (64)	81 (46)	0.456
Radiotherapy			
Yes	13 (24)	57 (22)	
No	41 (56)	201 (78)	0.751
Stage			
1	12 (22)	63 (24)	
2	10 (19)	56 (22)	
3	15 (28)	71 (28)	
4	17 (31)	68 (26)	0.865
Age			
<60	39 (72)	169 (66)	
≥60	15 (28)	89 (34)	0.341
LDH			
Normal	24 (48)	121 (47)	
High	26 (52)	136 (53)	0.905
Extranodal			
<2	38 (70)	182 (71)	
≥2	16 (30)	76 (29)	0.980
Performance			
≥70	42 (78)	231 (86)	
<70	12 (22)	37 (14)	0.148
IPI			
0-1	24 (46)	15 (45)	
2	12 (24)	56 (22)	
3	9 (18)	50 (19)	
4-5	6 (12)	37 (14)	0.942

**Table 6:** Multivariate analysis of prognostic factors significant in univariate analysis results.

	Hazard ratio	*P* value	95% confidence interval
Advanced age	**2.52**	**0.002**	**1.40**–**4.53**
Advanced stage	**2.37**	**0.038**	**1.05**–**5.38**
Low performance	**2.45**	**0.011**	**1.23**–**4.87**
High LDH	**2.48**	**0.013**	**1.20**–**5.09**
Extranodal ≥2 sites	0.68	0.276	0.34–1.36
Non-GC subgroup	1.62	0.103	0.91–2.89
Primary extranodal disease	1.11	0.824	0.44–2.83
